# Vitamin D Supplementation in Neonatal and Infant MIS-C Following COVID-19 Infection

**DOI:** 10.3390/ijms25073712

**Published:** 2024-03-27

**Authors:** Manuela Rizzi, Vincenzo Avellis, Alessandro Messina, Chiara Germano, Elena Tavella, Valentina Dodaro, Raffaele Vitale, Alberto Revelli, Paolo Zola, Simonetta Picone, Pier Michele Paolillo, Vito Mondì, Bianca Masturzo, Paolo Manzoni, Pier Paolo Sainaghi

**Affiliations:** 1 Department of Health Sciences (DiSS), Università del Piemonte Orientale (UPO), 28100 Novara, Italy; 2 IRCAD (Interdisciplinary Research Center of Autoimmune Diseases), Università del Piemonte Orientale (UPO), 28100 Novara, Italy; 3 School of Medicine, University of Turin, 10124 Turin, Italy; 4 Sant’Anna Hospital, Department of Surgical Sciences, University of Turin, 10126 Turin, Italybianca.masturzo@aslbi.piemonte.it (B.M.);; 5 Department of Maternal, Neonatal and Infant Medicine, University Hospital “Degli Infermi”, 13875 Ponderano, Italy; 6 Neonatology and Neonatal Intensive Care Unit, Policlinico Casilino, 00169 Rome, Italy; 7 Department of Translational Medicine (DiMeT), Università del Piemonte Orientale (UPO), 28100 Novara, Italy

**Keywords:** SARS-CoV-2, COVID-19, multisystem inflammatory syndrome in children (MIS-C), vitamin D, immunity

## Abstract

To date, the SARS-CoV-2 pandemic still represents a great clinical challenge worldwide, and effective anti-COVID-19 drugs are limited. For this reason, nutritional supplements have been investigated as adjuvant therapeutic approaches in disease management. Among such supplements, vitamin D has gained great interest, due to its immunomodulatory and anti-inflammatory actions both in adult and pediatric populations. Even if there is conflicting evidence about its prevention and/or mitigation effectiveness in SARS-CoV-2 infection, several studies demonstrated a strict correlation between hypovitaminosis D and disease severity in acute COVID-19 and MIS-C (multisystem inflammatory syndrome in children). This narrative review offers a resume of the state of the art about vitamin D’s role in immunity and its clinical use in the context of the current pandemic, specially focusing on pediatric manifestations and MIS-C. It seems biologically reasonable that interventions aimed at normalizing circulating vitamin D levels could be beneficial. To help clinicians in establishing the correct prophylaxis and/or supportive therapy with vitamin D, well-designed and adequately statistically powered clinical trials involving both adult and pediatric populations are needed. Moreover, this review will also discuss the few other nutraceuticals evaluated in this context.

## 1. Introduction

Severe acute respiratory syndrome coronavirus 2 (SARS-CoV-2) is a positive-sense, single-stranded RNA virus first described in December 2019 as the etiological agent responsible for a new coronavirus disease (COVID-19). To date, it is well accepted that COVID-19 displays highly heterogeneous clinical manifestations, ranging from an asymptomatic or paucisymptomatic disease to a life-threating condition characterized by features of interstitial pneumonia, acute respiratory distress syndrome (ARDS), and severe multiorgan failure [1,2,3,4]. In particular, those patients developing the most severe illness, which often results in death, have been reported to usually experience the so-called cytokine storm, a disproportional and dysregulated inflammatory response from both innate and adaptive immune cells, finally resulting in the systemic spread of large amounts of proinflammatory mediators, leading to severe organ dysfunctions [2,5,6]. 

**Figure 1:**
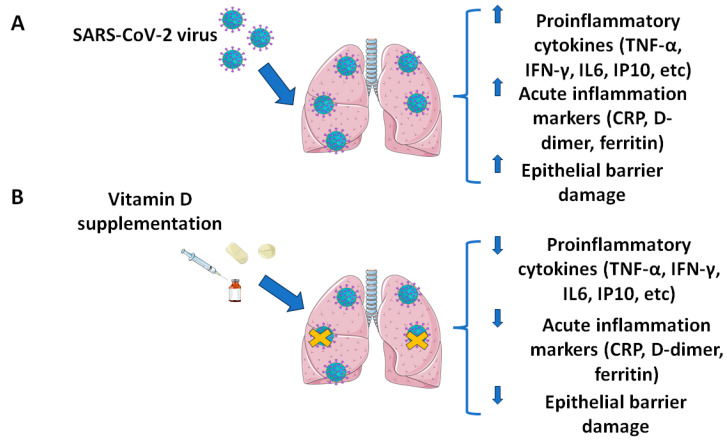
(**A**) Pathogenic mechanism of SARS-CoV-2 infection; (**B**) effect of vitamin D supplementation toward SARS-CoV-2 infection. The figure was partly generated using Servier Medical Art, provided by Servier, licensed under a Creative Commons Attribution 3.0 unported license (https://creativecommons.org/licenses/by/3.0/).

Considering that infection spread mainly relies on airborne transmission, and that the ACE2 (angiotensin-converting enzyme 2) receptor, the most common route of viral entry, is expressed by the epithelium lining the airways, COVID-19 symptoms are mainly represented by respiratory manifestations. Nevertheless, since the beginning of the pandemic, clinicians also observed that SARS-CoV-2 induced tissue damage in other body districts. Those clinical manifestations encompassing the expected respiratory district could be mainly explained by the high viral tropism toward the ACE2 receptor, the expression of which is not limited to the respiratory epithelium, but has been reported also in other extrapulmonary districts, such as the gut, kidneys, and cardiovascular and nervous systems [1,2,3,4]. In addition, the abnormal proinflammatory response observed in the most critical patients, characterized by a sustained increase in several cytokines, such as interleukin (IL) -4, IL-6, IL-8, IL-10, MCP-1 (monocyte chemoattractant protein-1), G-CSF (granulocyte colony-stimulating factor), TNFα (tumor necrosis factor α), IFNγ (interferon γ), and IP10 (IFN-inducible protein 10), as well as in other acute inflammation markers such as CRP (C reactive protein), D dimer, and ferritin, further contributes to the diffuse multi-organ damage (Figure 1A) [1,2,3,4,6,7].

Moreover, there is huge evidence that this disease mainly affects the adult population, with the most severe clinical presentations observed in the elderly and in high-risk subpopulations (i.e., immunocompromised and/or comorbid populations). Once again, the increased prevalence of COVID-19 in these fragile populations has been supposed to be related to ACE2 expression, which is known to be positively correlated to chronic comorbidity, as well as age, especially at the pulmonary level. Moreover, it should be considered that aging, immunosuppression, and chronic diseases are all conditions associated with immunological changes, such as alterations in IFNγ signaling and a decrease in specific lymphocyte populations (i.e., CD4^+^, CD8^+^ T cells, and naïve B cells), which are known to exacerbate the COVID-19 pathogenic process [7,8,9,10,11].

In contrast to what normally happens with virally driven respiratory infections, which are known to affect preferentially the children, since the beginning of the pandemic, SARS-CoV-2 showed a different trend. Children appear to be less affected by acute COVID-19 compared to adults, and most of the laboratory confirmed pediatric infections resulted in an asymptomatic or mild disease presentation, while severe illness appears to be an uncommon occurrence [8,10,11,12,13,14,15]. The most common explanation for this unexpected disease trajectory is based on age-specific differences in immune responses (i.e., stronger and more rapid innate as well as more naïve cellular immune responses in children compared to adults, the lower ability of activated innate immune cells to reach deep tissues in children compared to adults, and a higher prevalence of seasonal coronavirus infections, possibly resulting in “trained immunity”), lower mature ACE2 receptor expression in children’s respiratory system, and a lower prevalence of comorbidities. Furthermore, as highlighted by a recent review, the SARS-CoV-2-specific antibody response differs between the two age groups, with children showing the highest levels of anti-COVID-19 IgG and neutralizing antibody titers compared to adults, an immunological signature accounting for the observed reduced viral load and faster virus clearance in this population, further supporting the assumption of a quicker and more effective humoral response in children rather than in adults. Last but not least, it has been observed that in pediatric patients, their immune response to acute SARS-CoV-2 infection is characterized by lower amounts of proinflammatory cytokines, as the involved mechanism is more skewed toward a Th2-mediated response [7,8,13,15,16,17].

It is worth of note that, since the beginning of the pandemic emergency, the etiologic agent is continuously evolving in order to better escape host immune responses. As observed also for other RNA viruses, SARS-CoV-2 viral evolution is mainly driven by the ecological dynamics in host reservoirs and by the virus-specific mutation rate, finally resulting in the sequential appearance of different viral strains, showing different transmissibility and severity. Natural selection acts on these variants, resulting in fixing advantageous mutations, allowing the spread of those viral strains displaying better transmissibility and fitness in the final host [18]. This evolutionary process resulted in the acquisition of new infection routes, such as the ACE2/TMPRSS2 independent pathway described for the Omicron variant. In fact, to successfully infect its host, this viral strain takes advantage from the cleaving activity of endosomal cathepsins, thus expanding the number of potential cellular targets allowing its entry. This new viral strategy for entry is hypothesized to explain the observed higher transmissibility of this variant even in the pediatric population [15].

Considering the shortage of effective drugs able to fight COVID-19 infection, it is not surprising that nutritional supplements as well as remedies coming from traditional medicine have gained interest during the pandemic. Moreover, it is worth noting that the available pharmacological interventions are generally intended for adult use, while data about their effectiveness and safety in the pediatric population are limited. Thus, the use of nutritional supplementation to boost immune responses appears reasonable, especially in young populations. This narrative review will thus offer a resume of the current clinical evidence about the use of nutraceutical interventions in support of COVID-19 treatment, with a special focus on vitamin D, which appears to be the most promising one.

## 2. Multisystem Inflammatory Syndrome in Children (MIS-C)

Since April 2020, reports of children presenting with severe hyperinflammatory manifestations after COVID-19, resulting in a critical illness requiring hospitalization, started to appear. This acute condition, generally developing 3–6 weeks after SARS-CoV-2 infection, has been defined as “pediatric multisystem inflammatory syndrome temporally associated with COVID-19” (PIMS-TS) by the Royal College of Pediatrics and Child Health (RCPCH) in UK and “multisystem inflammatory syndrome in children” (MIS-C) by the World Health Organization (WHO) and by the US Centers for Disease Control and Prevention (CDC) [8,19,20,21].

To date, there is mounting evidence that a small minority of children infected with SARS-CoV-2 later develop MIS-C, especially in those regions where the COVID-19 burden was higher [16,19,21,22]. According to the CDC diagnostic criteria, MIS-C patients are younger than 21 years, present with fever and laboratory evidence of multisystem involvement (two or more domains involved), and need hospitalization in the absence of an alternative diagnosis. Moreover, most MIS-C patients generally test positive to COVID-19 or anti-SARS-CoV-2 antibodies [11,22,23,24,25,26,27]. From a clinical point of view, most MIS-C patients show abnormalities in inflammation markers, such as the elevated C-reactive protein (CRP), procalcitonin, erythrocyte sedimentation rate (ESR), D-dimer, lactic acid dehydrogenase, and ferritin [22,23,24,25,26,28,29,30,31,32,33]. Since one of the most common features of MIS-C is cardiovascular dysfunction (including coronary artery dilatation, depressed ventricular function, arrythmias, pericarditis, valvulitis, and more rarely aneurysms), elevated cardiac enzymes are also a common laboratory finding. Furthermore, in addition to cardiac involvement, the gastrointestinal domain is usually affected, even if, in the latter case, imaging results are often nonspecific [11,20,22,24,25,29,30,33,34].

Many of the above-mentioned MIS-C clinical features are also common to other hyperinflammatory conditions, such as Kawasaki disease (KD) and macrophage activation syndrome (MAS), making the differential diagnosis challenging [13,20,24,26,27]. Despite the symptoms overlapping mainly with Kawasaki disease, there are some epidemiologic differences, helping clinicians in differentiating between the two conditions. A summary of the currently available diagnostic criteria for MIS-C [19,24,25,35] and KD [36,37,38,39] is shown in Table 1. Moreover, it should be noted that the available evidence accounts for an older age of MIS-C patients compared to KD (median 7–11 years vs. < 5 years of age), and for a higher prevalence of the disease in children of Hispanic and African ancestry compared to the well-known predominance of Kawasaki disease in children of Asian descent. Moreover, the common gastrointestinal involvement observed in MIS-C is less frequent in Kawasaki disease-affected children [11,13,24,25,26,29,33,34,40].

Furthermore, it is noteworthy that MIS-C results from an abnormal immune response to acute SARS-CoV-2, supporting the current view of its post-infectious nature [13,16,24,34,41,42]. Even if the MIS-C pathogenic mechanism is still unknown, it is strongly supposed to be an autoimmune vasculitis, in which higher anti-SARS-CoV-2 IgG antibodies are associated with a cytokine storm characterized by increased levels of pro-inflammatory cytokines (i.e., TNFα, IL1β, IL6, IL8, IL18) and with increased coronary disease biomarkers, thus resulting in diffuse endothelial damage through immune complexes and complement pathways’ activation. The MIS-C cytokine storm displays some overlapping characteristics with MAS, but also in this case there are some differences (i.e., a different degree of inflammatory markers) helping clinicians in differentiating between the two clinical conditions [13,24,43].

To date, MIS-C incidence is not yet known, but it seems to be rare (<1% of COVID-19 infected children or 2:10^5^ individuals aged < 21 years, according to different reviews) [23,25,44] and, interestingly, even if MIS-C complications could be severe, requiring pediatric intensive care support, the overall prognosis of such patients appears to be positive, with a large majority of patients experiencing a full clinical recovery [23,25,34]. Furthermore, it is noteworthy that in several studies it has been observed a decrease in disease incidence during the period of the Omicron variant prevalence [28,45]. In spite of such comforting evidence, to date no universally agreed therapeutic approaches are available for MIS-C, and the clinical presentation and disease evolution still drive pharmacological interventions. Due to the observed similarities with KD, especially for what concerns cardiac dysfunction, therapeutic approaches are usually extrapolated from such condition, with intravenous immunoglobulin (IVIG) infusion as first-line therapy, usually in association with steroids and anticoagulants (in those cases where these drugs are clinically contraindicated). In those clinical presentations refractory to first-line therapies, anti-cytokine approaches (i.e., IL1 blockade (with anakinra or cankinumab) and/or IL6r blockade (with tocilizumab)) have proven to be useful [16,21,24,25,26,30,32,34,40,44,46]. 

Given the post-infectious nature of MIS-C and the wide spectrum of its symptoms, it is noteworthy that a prompt recognition and a timely treatment, limiting hyperinflammatory responses and subsequent organ damages, are essential to favor a good outcome. The observed lack of a specific therapeutic strategy for this emerging disease fostered the research of effective add-on approaches to improve currently available drug treatments’ effectiveness. To reach such objectives, many researchers have focused their attention on patients’ nutritional needs, as there is growing evidence supporting the role of a balanced nutrition in supporting immune functions in both adults and children [47,48,49,50,51,52,53,54,55,56]. 

## 3. Nutrition and Immunity against SARS-CoV-2 Infection

A healthy lifestyle along with a balanced diet are essential to support the individual immune system’s functioning and the overall well-being. It is known that malnutrition increases individual susceptibility to infectious diseases, which in turn contributes to malnutrition through the development of a pathological vicious cycle [49,52,57,58]. Malnutrition effects on immune function are evident if we consider that both innate and adaptive immune responses rely on specific cellular populations that need to quickly respond to the potentially noxious stimulation, resulting in an increased need in nutritive substrates, such as glucose, amino acids, and fatty acids, to be converted into energy [49,53,55,57,59]. This strict relation between nutrition and immune function is even more important in children, where acute and chronic malnutrition accounts for the most severe clinical manifestations, as malnourished children are more susceptible to infections, which in turn result in an increased nutritional need as well as in an increased nutrients’ loss via cytokine-driven pathways. Moreover, childhood represents a period of each individual’s life in which the immune system is still developing and acquiring its full protective potential; for this reason, a diverse and balanced diet since the first years of life plays a key role in promoting immune tolerance and in preventing allergic diseases, as well as in supporting the proper development of immunity by modulating immune cells’ maturation as well as microbiome composition [51,52].

Furthermore, there is evidence supporting the role of nutrient intake (intended as directly derived from the diet or acquired through dietary supplements) in modulating both immune cells’ maturation and response to inflammation by acting through epigenetic mechanisms and by affecting the host’s intestinal and pulmonary microbiota composition [47,51,54,56,60]. A key role in this context is played by vitamins (i.e., vitamin A, vitamin C, and vitamin D) and oligoelements (i.e., zinc, selenium), which deficiency has been shown to affect, to a various extent, immune functions. Moreover, also dietary supplements, such prebiotics and probiotics, are known to be important in supporting immune defenses, as they directly affect microbiota composition and functions [19,47,50,53,54,56,59,60,61].

Considering the well-recognized role of nutrition in improving immune function, it is not surprising that, during the pandemic and especially during the confinement phases, the consumption of dietary supplements showed a great increase all over the world. Among the most chosen over-the-counter supplements were vitamins (i.e., vitamin C, vitamin D), oligoelements (i.e., zinc, selenium), hormones (i.e., melatonin), compounds with an immunomodulatory effect (i.e., lactoferrin), and herbal products belonging to Chinese and Indian traditional pharmacopeia [62,63,64,65,66,67]. 

Furthermore, due to the lack of effective and targeted pharmacological treatments for COVID-19, nutrition has been regarded as a potential supportive approach in preventing and/or mitigating SARS-CoV-2 infection outcomes, accounting for the investigation, in different clinical trials and observational studies, of some nutritional supplements. Unfortunately, this research resulted in conflicting results, mainly depending on the different study design (i.e., timing of supplement administration, supplement dose, disease severity, age of the participants), thus preventing the definition of specific guidelines.

Among the different dietary supplements tested during the pandemic, we can take as an example the case of lactoferrin, a milk-derived glycoprotein with antiviral and immunomodulatory activity [68,69,70] that has been shown to be able to prevent SARS-CoV-2 infection [70] and to accelerate clinical recovery [71,72] when administered just following infection confirmation. On the other hand, when it was administered in more severe patients, following their hospitalization due to the worsening of their symptoms, such supplement failed to show any positive effect [73,74]. Such conflicting results could be explained considering the used lactoferrin formulation (protected vs. non-protected against gastric digestion) and, especially, by the timing of administration, as neutral/negative results were obtained in those trials in which the supplement was administered as an add-on to the standard of care treatment, based on high doses of corticosteroids and heparin that could have masked, at least partially, lactoferrin activities.

Similar conflicting results have been obtained also with vitamin C (ascorbic acid), a nutritional supplement largely used in the prevention and treatment of common colds and other seasonal respiratory infections. In the first phases of the pandemic, ascorbic acid has been investigated as a potential adjuvant therapy against COVID-19, as this vitamin is known to promote immune functions by reducing oxidative stress, modulating cytokine production, and promoting B- and T-lymphocytes’ proliferation and differentiation, as well as to improve epithelial and endothelial barrier functions [67,75,76,77]. Once again, the results were inconclusive, mainly because of the large heterogeneity in the intervention design. While several studies investigating the effect of the intravenous infusion of high doses of ascorbic acid in severely ill patients showed promising results [78,79,80,81], others investigating the effects of high doses of vitamin C administered orally or intravenously to both non-severe and severe patients failed to show any significant benefit [82,83,84,85].

Also, the multifunctional hormone melatonin (N-acetyl-5-mehoxytryptamine), the secretion of which is maximal in children and adolescents, has been tested as a potentially useful compound in COVID-19 treatment [67]. Melatonin supplementation showed some promising results, even if no conclusive clinical evidence from specific trials is available. This hormone is known to interact with cortisol as well as with immune and inflammatory pathways altered in SARS-CoV-2 infection. Furthermore, it is also involved in reducing mitochondrial oxidative stress at the lung level while assuring systemic antioxidant protection [67,86,87]. The preliminary data from the ongoing clinical trials on melatonin supplementation show a reduction in the CRP plasma level but not in clinical endpoints as survival and mechanical ventilation duration in intubated patients exposed to the hormone [88], but also an improvement in clinical findings and a more rapid disease resolution in hospitalized mild to moderate COVID-19 patients treated with standard-of-care therapies [89]. Its potential effectiveness as an add-on compound to standard therapy has also been supported by a recent multidrug repurposing study, showing a 28% reduced likelihood of infection in the general population [90]. This evidence, even if promising, is preliminary and derived from small monocentric cohorts and needs to be confirmed on lager multicenter studies to make any suggestion for clinical use.

To further complicate the situation, many of the studies focusing on nutritional interventions in the context of the ongoing pandemic were conducted on the adult population, thus preventing clinicians from inferring specific conclusions to be applied to the pediatric population.

The only exception is represented by vitamin D, the immunomodulatory role of which has been investigated both in adults and children infected with SARS-CoV-2.

## 4. Vitamin D and Immunity against SARS-CoV-2 Infection

Vitamin D is a fat-soluble pre-hormone and its main physiological role is related to calcium and phosphorus homeostasis. To date, it is known that its biological activity is wider than previously thought, as this compound is involved in many extra-skeletal functions and its deficiency/insufficiency is related to many diseases (i.e., hypertension, autoimmune diseases, metabolic syndrome, etc.). Furthermore, hypovitaminosis D has been associated with an increased susceptibility to respiratory infections and, consequently, to an increased SARS-CoV-2 infection risk [85,91,92,93,94,95,96,97].

Vitamin D comes from three potential sources: UVB-dependent endogenous synthesis at skin level, diet, and nutritional supplements in the form of cholecalciferol (vitamin D_3_, of animal origin) or ergocalciferol (vitamin D_2_, of plant origin). Independently from its origin, at the body level, to become biologically active, vitamin D needs to be metabolized to 1,25di-hydroxy-vitamin D (calcitriol) by two consecutive hydroxylation steps at the hepatic and renal level. The biologically active molecule then interacts with a specific receptor (VDR) ubiquitously expressed in nucleated cells. Considering that VDR is a ligand-dependent transcription factor, it is worth noting that calcitriol has been demonstrated to regulate, directly or indirectly, over 1000 human genes [92,93,94,95,96,97,98,99,100,101,102]. 

Importantly, even if circulating vitamin D levels mainly depend on the kidney hydroxylation step, also other extra-renal tissues express the unique 1α-hydroxylase CYP27B1, the enzyme needed to assure the final activating hydroxylation, thus allowing vitamin D autocrine and paracrine hormonal actions. Among the “non-canonical” targets of vitamin D, the most studied is the immune system, where it exerts both immunomodulating and anti-inflammatory actions [92,93,94,98,101,103].

In the immune system, it has been demonstrated that CYP27B1 expression is tightly regulated by different inputs. In particular, it has been observed that hydroxylase transcription in immune cells is regulated by signals coming from the invading pathogen (i.e., bacterial lipopolysaccharide, ligands of the toll-like receptor pathways, pathogens, and T cell-derived proinflammatory cytokines), leading to the local generation of the active compound. The local synthesis of calcitriol is important in T lymphocytes’ functioning, as it regulates both their proliferation and cytotoxic responses as well as their migration and development, especially for memory T cells, which are characterized by a higher VDR expression [102].

The vitamin D immunomodulatory action mainly relies on its interaction with VDR, which subsequently binds the vitamin D response element (VDRE) located upstream the promoter region of different actors of the immune response against threats of bacterial and viral origin, such as cathelicidins and defensins [92,93,94,98,101,102,103,104,105,106,107]. Vitamin D modulates both the innate and acquired immune responses, as VDR expression varies according to immune cells’ activation status (i.e., VDR expression is increased upon B- and T-cell activation, while it is decreased in differentiating monocytes). When vitamin D binds to VDR, it has been proven to affect different intracellular signaling pathways related to the immune defense, such as the NF-kB and glucocorticoid receptor-mediated ones, as well as to suppress the activation of antigen- specific CD8 T cells and to stimulate the production of Treg cells. Moreover, vitamin D also exerts anti-inflammatory actions by downregulating the production of different pro-inflammatory cytokines (especially IL-6 and TNF-α) and by increasing the expression of anti-inflammatory mediators, such as IL-10, as well as by inducing tolerogenic components of dendritic cells populations (Figure 1B) [93,95,98,100,101,102,103,104,106,108,109,110].

Last but not least, it should be remembered that vitamin D not only drives immune responses by regulating immune cells’ functioning, but is also involved in maintaining the integrity of the epithelial barriers and in the regulation of intestinal and respiratory microbiota, thus further contributing to the host’s defenses toward invading pathogens. In particular, the vitamin D/VDR complex is known to modulate both the way in which pathogens are recognized (e.g., by influencing the pathogen recognition receptors) and the host’s responses (e.g., NF-kB- and IL1β-driven downstream responses). In addition to directly regulating the transcription of cathelicidins and defensins, the vitamin D/VDR complex is also able to regulate the cytosolic components of the pathogen recognition system, such as the inflammasome-driven response [103,111].

Considering that hypovitaminosis D has been associated with an increased risk of respiratory infection and that vitamin D supplementation has proven to have anti-inflammatory effects in the lungs [107,112,113,114], since the beginning of the pandemic, several studies have tried to address the relationship between vitamin D status and COVID-19 infection and severity.

Actually, it is well accepted that hypovitaminosis D is a risk factor for COVID-19 as many ecological studies have highlighted a greater prevalence of the disease in those populations showing lower vitamin D levels [105,115,116,117,118,119,120]. Moreover, even on a smaller scale, when considering single cohorts of patients, basal low vitamin D levels are an independent predictor of COVID-19 severity [109,121,122,123,124]. 

Considering that severe COVID-19 evolution is characterized by a hampered immune response culminating in the so-called “cytokine storm” and that vitamin D has proven to be effective in mitigating hyperinflammation and ARDS, as well as in promoting anti-viral responses and in increasing pulmonary surfactant production, several clinical trials and observational studies have been conducted to evaluate the effectiveness of vitamin D supplementation in preventing SARS-CoV-2 infection and in mitigating COVID-19 evolution [93,103,105,125,126].

As already observed for the other dietary supplements that have been tested as adjuvant therapies in COVID-19, the clinical research focusing on vitamin D showed mixed results, mainly depending on the large heterogeneity in the studies’ design (especially in terms of vitamin D dose, formulation (biologically active vs. inactive metabolite), frequency of administration, and supplementation duration). Several trials and observational cohort studies showed that vitamin D supplementation was beneficial against SARS-CoV-2 infection and significantly reduced the need of intensive care in COVID-19 hospitalized patients [113,127,128], while others failed to highlight any significant improvement in disease outcomes [108,129,130]. The results coming from the most relevant clinical studies dealing with the evaluation of vitamin D supplementation in improving or mitigating COVID-19 clinical evolution are summarized in Table 2.

As stated before, vitamin D supplementation’s beneficial effects have been studied also in the pediatric population, where it has been proven to reduce the rate of infections and to prevent autoimmune manifestations, all conditions for which hypovitaminosis D is considered a risk factor [94,131,132,133,134]. Considering the existing evidence on the usefulness of vitamin D supplementation in the pediatric population [94,133,134] and the proven susceptibility of this specific population to SARS-CoV-2 infection, especially with the emergence of novel variants such as Delta and Omicron, which seem to have a higher incidence and an increased hospitalization rate in children [135,136,137], it is not surprising that there was a growing interest in clinical studies (clinical trials, but also observational studies) focused on vitamin D supplementation in children as a potential adjuvant therapy.

As already observed in the adult population, also in the pediatric age, low vitamin D levels are associated with COVID-19 severity [131,132,138,139], thus suggesting the effectiveness of vitamin D replacement in supporting immune function against SARS-CoV-2 infection in children, as demonstrated by Zurita-Cruz and colleagues in a randomized controlled clinical trial where vitamin D supplementation reduced the risk of COVID-19 adverse outcomes in a pediatric cohort without showing any serious safety issues [140] (Table 2).

**Table 2:** Summary of the most relevant clinical studies investigating vitamin D effectiveness in COVID-19 management.

Country	Study Design	Study Objective	Patients Description	Number of Patients Enrolled	Intervention	Main Results	Reference
Mexico	Open label, randomized, controlled, single-blind clinical trial	Efficacy and safety of vitamin D supplementation in pediatric patients hospitalized due to moderate COVID-19	Pediatric patients (1 month–17 years old) requiring hospitalization due to moderate COVID-19 and supplemental oxygen	45 randomized, 20 (17 analyzed) to the intervention arm, 25 (analyzed 21) to the control arm	1000 IU/day (children aged < 1 year) or 2000 IU/day (children aged 1–17 years) for a minimum of 7 and a maximum of 14 days in addition to the standard of care	The study was stopped due to ethical reasons *, but until its end, vitamin D supplementation seemed to be able to decrease the risk of COVID-19 progression and death	[140]
Croatia	Single center, open label, randomized clinical trial	Effectiveness of a daily supplementation of vitamin D during ICU stay in improving clinical outcomes	Adults admitted to ICU due to COVID-19 with a need for invasive or non-invasive respiratory support and vitamin D levels < 50 nmol/L	152 randomized, 75 to the intervention arm, and 77 to the control arm	Daily 10,000 IU cholecalciferol (oral suspension) administered orally or via gastric tube for 14 days during ICU stay in addition to the standard of care	No statistically significant effect in reducing the number of days spent on respiratory support nor on the secondary outcomes (all-cause mortality on days 14, 28, 60; clinical improvement at day 28; number of days spent in ICU, number of days spent in hospital, bacterial superinfections, neutrophil/lymphocyte ratio, CRP levels, PaO_2_/FiO_2_ ratio, D-dimer, ferritin, fibrinogen and procalcitonin levels)	[108]
Spain	Prospective, nonselected, observational cohort study	Effects of calcifediol supplementation on COVID-19- related outcomes	Adults hospitalized due to moderate–severe COVID-19	838 (intention to treat), 447 receiving the intervention, and 391 not treated (control group)	Oral calcifediol in soft capsules (0.532 mg at admission, 0.266 mg on day 3, 7, 15, and 30) in addition to the standard of care	Statistically significant reduction in ICU admission and mortality rates	[113]
Spain	Retrospective cohort study	Effectiveness of cholecalciferol or calcifediol supplementation in improving COVID-19 clinical outcomes	Adults affected by COVID-19 (population-based study)	All individuals living in Barcelona–Central Catalonia district (4.6 million)	Eligible patients were identified as patients receiving therapeutic formulations containing > 0.25 mg of cholecalciferol or calcifediol per dose	In a population-based setting, cholecalciferol or calcifediol supplementation allowing the restoration of plasma vitamin D levels ≥ 30 mg/mL was associated with better COVID-19 outcomes (SARS-CoV-2 infection risk, disease severity, and associated mortality)	[127]
Spain	Parallel arms, pilot, randomized, open label, double-masked clinical trial	Effectiveness of early calcifediol administration in reducing ICU admission rate and mortality rate	Adults with moderate COVID-19 requiring hospitalization	76 randomized, 50 to the intervention arm, and 26 to the control arm	Oral calcifediol in soft capsules (0.532 mg at admission, 0.266 mg on day 3 and 7 and then weekly until discharge or death) in addition to the standard of care	Early high dose calcifediol administration following the proposed therapeutic schedule significantly reduced the need of ICU admission in patients hospitalized due to proven COVID-19 disease. Furthermore, all the patients belonging to the intervention arm survived	[128]
Spain	Multicenter, international, randomized, open label clinical trial	Effectiveness of an oral bolus of high-dose cholecalciferol administered at hospital admission in modifying moderate–severe COVID-19 clinical outcomes	Adults with moderate–severe COVID-19 requiring hospitalization	548 randomized, 277 (274 analyzed) to the intervention arm, and 271 (269 analyzed) to the control arm	100,000 IU cholecalciferol (single oral bolus) in addition to the standard of care	No statistically significant effects of the intervention in reducing the median length of hospitalization, the ICU admission rate, nor the mortality rate	[129]
Brazil	Multicenter, double blind, randomized, placebo-controlled clinical trial	Effect of a single high dose of vitamin D_3_ on the duration of hospitalization	Adults hospitalized due to moderate–severe COVID-19	240 randomized, 120 to the intervention arm, and 120 to the control arm	200,000 IU vitamin D_3_ (single oral bolus) in addition to the standard of care	No statistically significant effect in reducing the hospital stay length nor in reducing the in-hospital mortality rate, the ICU admission rate, nor the need for mechanical ventilation support	[130]

* The study was stopped due to ethical reasons as the initial data demonstrated that all the enrolled subjects had a baseline vitamin D concentration lower than the commonly accepted limit used for defining vitamin sufficiency. Considering the ongoing pandemic emergency, the ethical committee and the investigators decided to stop the trial recruitment and to offer vitamin D supplementation to all patients being hospitalized due to COVID-19.

Regarding the consequences of SARS-CoV-2 infection in children, it should be remembered that during the pandemic progression, it has been observed a rise in MIS-C cases [16,23,41]. Considering the proven relationship between low vitamin D levels and COVID-19 severity both in adults and children, it is not surprising that vitamin D status was investigated also in relation with this disease’s manifestation. 

As expected, also in MIS-C patients, it has been observed a correlation between hypovitaminosis D and disease severity [23,141]. The most plausible explanation of this association resides in the proven immunomodulatory effects of vitamin D, since it is well-accepted that MIS-C pathophysiology is related to a dysregulated immune response [23,31,141]. Moreover, it should be kept in mind that MIS-C patients often show cardiac involvement and that, in several epidemiological studies, vitamin D deficiency has been associated with cardiovascular diseases due to its critical role in endothelial homeostasis and function [142,143,144]. Even if the available literature about vitamin D in MIS-C is limited, hypovitaminosis D has been observed in severe MIS-C patients, showing cardiovascular involvement [23,141], thus supporting further studies on this topic.

## 5. Conclusions

At the time of writing, we are facing the third year of the COVID-19 pandemic, and a huge amount of scientific evidence is accumulating from both experimental and clinical studies. Moreover, it is of note that during the past years, clinical features of the disease have changed, according to the emergence of new viral variants. Despite this evolution in the disease’s manifestations, observations about the role of nutrition appear still relevant, especially in light of the current shortage in targeted and effective therapeutic approaches, especially in low-income countries. 

Even if the current evidence about nutrients’ role in SARS-CoV-2 infection prevention and in COVID-19 outcomes’ mitigation is inconclusive, due to the large heterogeneity in the study design and to the large number of under-powered studies, research in this field is worth of interest. In particular, vitamin D appears as a very promising candidate, both in the adult and pediatric population. 

In spite of the existing discordant thresholds used to define vitamin D deficiency/insufficiency and normality, a large body of evidence supports a correlation between hypovitaminosis D and SARS-CoV-2 infection severity, thus suggesting a potential adjuvant role of vitamin D supplementation in COVID-19 clinical management. This observation becomes even more relevant if we consider that an adequate supplementation of vitamin D has been proven beneficial in treating respiratory infections, where the immune cell infiltration and the hyperinflammatory response is mitigated by correcting the underlying hypovitaminosis D.

Even if, actually, there is no evidence explicitly supporting systematic vitamin D supplementation in all age ranges, the accumulating evidence from clinical trials and observational studies highlight an ancillary role of its supplementation in mitigating COVID-19 manifestations. Furthermore, it is worth noting that such recomforting observations are supported by in vitro data, highlighting vitamin D antiviral and antimicrobial actions as well as its ability to directly modulate immune cells’ behavior by reducing proinflammatory cytokine production as well as by increasing anti-inflammatory mediators’ expression.

According to some recent meta-analyses, vitamin D is a cheap and widely available nutritional supplement, the administration of which is relatively safe and could protect from the most detrimental consequences of SARS-CoV-2 infection and thus could represent a valuable intervention tool especially in low-income countries and in all those situations where a full access to medical treatments is limited.

Considering the lack of safety issues highlighted by the current available trials on both adult and pediatric populations, it appears reasonable that vitamin D supplementation to correct underlying, sometimes latent hypovitaminosis D could represent an easily implementable intervention aimed to boost immune responses, especially in high-risk populations. Furthermore, considering the rise in MIS-C cases, especially in those regions where the COVID-19 burden was higher, such a quick, safe, and generally high-compliance intervention could represent a valuable tool in managing this already little-understood clinical condition, for which effective and targeted therapeutic interventions, especially intended for pediatric use, are still lacking.

For these reasons, further investigations about vitamin D effectiveness as a COVID-19 and MIS-C severity marker as well as vitamin D’s supplementation role in these patients are warranted. In particular, specifically designed clinical trials focusing on infants and children would be essential to identify the correct nutritional approach in a population with peculiar needs in terms of macro- and micronutrients.

## Figures and Tables

**Table 1:** Diagnostic criteria for MIS-C and KD according to the most relevant clinical guidelines. Abbreviations: MIS-C = Multisystem Inflammatory Syndrome in Children, CDC = US Centers for Disease Control and Prevention, WHO = World Health Organization, RCPCH = Royal College of Pediatrics and Child Health, AHA = American Heart Association, JCS/JSCS = Japanese Circulation Society/Japanese Society for Cardiovascular Surgery, ECHO = echocardiography, NT-proBNP = N-terminal pro-B type natriuretic peptide, PT = prothrombin time, PTT = activated partial thromboplastin time, ESR = erythrocyte sedimentation rate, CRP = C-reactive protein.

MIS-C			Kawasaki Disease	
CDC	WHO	RCPCH	AHA	JCS/JSCS
Age < 21 years	Age 0–19 years	A child presenting with persistent fever, inflammation (neutrophilia, elevated CRP, and lymphopenia), and evidence of single or multiorgan dysfunction (shock, cardiac, respiratory, renal, gastrointestinal, or neurological disorders) with additional features ( listed in the guidance document). This may include children fulfilling full or partial criteria for Kawasaki disease	Fever lasting for ≥5 days	**At least five of the** **following**
**AND**	Fever lasting for ≥3 days	Exclusion of any other microbial cause, including bacterial sepsis, staphylococcal or streptococcal shock syndromes, and infections associated with myocarditis such as enterovirus (waiting for the results of these investigations should not delay seeking expert advice)	**AND four of the following:**	Fever
Fever ≥ 38 °C lasting for at least 24 h	**AND two of the following:**	SARS-CoV-2 testing may be positive or negative	Conjunctivitis (bilateral, bulbar, conjunctival injection without exudate)	Bilateral bulbar conjunctival injection
Laboratory evidence of inflammation	Rush or bilateral non-purulent conjunctivitis or muco-cutaneous inflammation signs (oral, hands, or feet)		Lymphadenopathy (cervical, often > 1.5 cm, usually unilateral)	Changes to the lips and oral cavity (reddening of lips, strawberry tongue, diffuse injection of oral and pharyngeal mucosae)
Clinical evidence of severe illness requiring hospitalization	Hypotension or shock		Rush (maculopapular, diffuse erythrodema, or erythema multiforme)	Rush (including redness at the site of Bacille Calmette–Guèrin (BCG) inoculation)
Multisystem organ involvement (≥2 among cardiac, renal, respiratory, hematological, gastrointestinal, dermatological, or neurological domains)	Features of myocardial dysfunction, pericarditis, valvulitis, or coronary abnormalities (including ECHO findings or elevated troponin/NT-proBNP)		Changes in lips or oral mucosa (red cracked lips, strawberry tongue, or diffuse erythema of oropharynx)	Changes in the peripheral extremities: Reddening of palms and soles, edema (initial stage) Periungual desquamation (convalescent stage)
**AND**	Evidence of coagulopathy (prolonged PT, PTT, elevated D-dimers)		Changes to extremities (erythema and oedema of palms and soles in acute phase and periungual desquamation in subacute phase)	Nonsuppurative cervical lymphadenopathy
No alternative plausible diagnosis	Acute gastrointestinal symptoms (diarrhea, vomiting, or abdominal pain)			**ALTERNATIVELY**
**AND**	**AND**			At least four of the above
Current or recent SARS-CoV-2 infection (positivity assessed by RT-PCR, serology, or antigen test) or exposure to a suspected or confirmed COVID-19 case within the 4 weeks prior to symptoms onset	Elevated inflammation markers (ESR, CRP, or procalcitonin)			**AND**
	**AND**			Exclusion of other febrile illnesses
	No other obvious microbial causes of inflammation (bacterial sepsis, staphylococcal or streptococcal shock syndromes)			**AND**
	**AND**			Coronary artery dilation (Z-score of internal coronary artery diameter ≥ 2.5 SD units or absolute diameter ≥ 3 mm (<5 years old) or ≥4 mm (≥5 years old))
	Evidence of COVID-19 (RT-PCR, antigen test, or serology positive) or likely contact with patients with COVID-19			

## Data Availability

Not applicable.
